# Age-specific reference values for low psoas muscle index at the L3 vertebra level in healthy populations: A multicenter study

**DOI:** 10.3389/fnut.2022.1033831

**Published:** 2022-12-16

**Authors:** Ming Kong, Ning Lin, Lili Wang, Nan Geng, Manman Xu, Shanshan Li, Wenyan Song, Ying Zhou, Yuetong Piao, Zuoqing Han, Rong Guo, Chao Yang, Nan Luo, Zhong Wang, Quanxiao Xu, Daimeng Shi, Wanchun Qiu, Junfeng Li, Eddie C. Cheung, Lei Ma, Yu Chen, Zhongping Duan

**Affiliations:** ^1^Beijing Municipal Key Laboratory of Liver Failure and Artificial Liver Treatment Research, Fourth Department of Liver Disease, Beijing Youan Hospital, Capital Medical University, Beijing, China; ^2^Department of Radiology, The First Hospital of Lanzhou University, Lanzhou, China; ^3^Department of Radiology, Beijing Youan Hospital, Capital Medical University, Beijing, China; ^4^Department of Gastroenterology and Hepatology, The Chinese People’s Liberation Army Rocket Force Characteristic Medical Center, Postgraduate Training Base of Jinzhou Medical University, Beijing, China; ^5^Department of Infection, Dalian Medical University, Dalian, China; ^6^Department of Radiology, The Second Hospital of Dalian Medical University, Dalian, China; ^7^Department of Infection, The Second Hospital of Dalian Medical University, Dalian, China; ^8^Diagnosis and Treatment Center of Hepatobiliary Diseases, Nanyang First People’s Hospital, Nanyang, China; ^9^Department of Infection, The First Clinical Medical School of Lanzhou University, Lanzhou, China; ^10^Department of Infectious Diseases, Institute of Infectious Diseases, The First Hospital of Lanzhou University, Lanzhou, China; ^11^Division of Gastroenterology, School of Medicine, University of California, Davis, Davis, CA, United States; ^12^Center for Digestive Disease, The Seventh Affiliated Hospital, Sun Yat-sen University, Shenzhen, China

**Keywords:** skeletal muscle mass, sarcopenia, computed tomography, lumbar 3 vertebra, reference values, healthy adults

## Abstract

**Background and aims:**

The progressive and generalized loss of skeletal muscle mass, strength and physical function is defined as sarcopenia. Sarcopenia is closely related to the prognosis of patients. Accurate diagnosis and adequate management of sarcopenia are crucial. The psoas muscle mass index taken at the third lumbar vertebra (L3-PMI, cm^2^/m^2^) is one of the established methods for evaluating skeletal muscle mass. However, the cutoff values of L3-PMI for diagnosis of sarcopenia are not yet to be clarified in Asian populations. We attempted to establish reference values for low L3-PMI that would be suitable for defining sarcopenia in the Northern Chinese population.

**Methods:**

This was a retrospective, multicenter cross-sectional study. A search of abdominal CT imaging reports was conducted in four representative cities in northern China. Transverse CT images were measured using the analysis software Slice-O-Matic. Low psoas muscle index was defined as the 5th percentile or mean-2SD of the study group.

**Results:**

1,787 healthy individuals in the study were grouped by age. The sex and number of people in each group were similar. L3-PMI had a negative linear correlation with age, and a strong correlation with the skeletal muscle index taken at the third lumbar vertebrae (L3-SMI, cm^2^/m^2^). The L3-PMI reference values in males were 5.41 cm^2^/m^2^ for 20–29 years, 4.71 cm^2^/m^2^ for 30–39 years, 4.65 cm^2^/m^2^ for 40–49 years, 4.10 cm^2^/m^2^ for 50–59 years and 3.68 cm^2^/m^2^ for over 60 years by using 5th percentile threshold. Similarly, the reference values in females were 3.32, 3.40, 3.18, 2.91, and 2.62 cm^2^/m^2^. When using mean-2SD as the reference, the values for each age group were 4.57, 4.16, 4.03, 3.37, and 2.87 cm^2^/m^2^ for males and 2.79, 2.70, 2.50, 2.30, and 2.26 cm^2^/m^2^ for females, respectively.

**Conclusion:**

We defined the reference values of age-specific low skeletal muscle mass when simply evaluated by L3-PMI. Further studies about the association of sarcopenia using these reference values with certain clinical outcomes or diseases are needed.

## Introduction

According to the GLIM consensus, malnutrition is defined as inadequate nutrient intake and absorption and varying degrees of acute or chronic inflammation, leading to altered body composition and diminished biological function. Skeletal muscle loss is the principal manifestation of altered body composition (1). The progressive and generalized loss of skeletal muscle mass, strength and physical function is defined and diagnosed in detail by sarcopenia. Sarcopenia can be divided into primary sarcopenia due to aging and secondary sarcopenia due to acute or chronic illness (2–4). According to the reports, sarcopenia is an independent predictor of longer hospital stay, increased incidence of infections and 12-month mortality post-liver transplantation (5). Therefore, accurate diagnosis and adequate management of sarcopenia are crucial.

Skeletal muscle mass, the main component of identifying sarcopenia, can be measured in by dual-energy x-ray absorptiometry (DXA), computed tomography (CT), magnetic resonance imaging (MRI) and bioimpedance analysis (BIA) (6). Given that CT is easy to obtain and has high accuracy, it has been used to determine the various skeletal muscle mass cutoff values in western countries (7). The psoas muscle mass index taken at the third lumbar vertebra (L3-PMI, cm^2^/m^2^) (the sum of the third cross-sectional areas of the right and left psoas/height squared) is one of the established methods for evaluating skeletal muscle mass (8). So far, some studies have evaluated the L3-PMI cutoff values for the diagnosis of skeletal muscle mass loss in healthy adults and in patients with chronic disease in Asian populations (9, 10). In these reports, the size of sample was relatively limited and the definition of L3-PMI was unified. The cutoff values of L3-PMI for diagnosis of sarcopenia are not yet to be clarified in Asian populations. Thus, we investigated the psoas muscle mass of the healthy Asian population in four representative cities in Northern China to explore the muscle mass distribution of the population.

In the present study, we investigate the correlation between L3-PMI with the skeletal muscle index taken at the third lumbar vertebrae (L3-SMI, cm^2^/m^2^), sex and age. According to the muscle mass distribution of the population, we attempted to establish reference values for low L3-PMI that would be suitable for defining sarcopenia in the Northern Chinese population.

## Materials and methods

### Study design and population

This was a retrospective, multicenter cross-sectional study. Between January 2016 and March 2021, there were 1,787 healthy adults included in the study from the following four representative cities in northern China: Beijing (*n* = 471), Dalian (*n* = 517), Lanzhou (*n* = 381) and Nanyang (*n* = 418). A comprehensive search of abdominal CT imaging reports was conducted in the Radiology Information System at Beijing Youan Hospital, Capital Medical University (Beijing, China), Nanyang First People’s Hospital (Nanyang, China), the Second Hospital of Dalian Medical University (Dalian, China), and the First Hospital of Lanzhou University (Lanzhou, China). The specific search keywords were “no obvious abnormality,” “liver cyst,” “liver hemangioma,” “gallbladder stone,” “kidney cyst,” and “kidney stone.”

The Inclusion criteria are as follows: (1) aged over 20 years old; (2) having an abdomen CT examination (including L3 level); (3) BMI ≥ 18.5kg/m^2^. The Exclusion criteria are as follows: (1) malignancy; (2) various chronic diseases, such as cardio-cerebrovascular diseases, chronic obstructive pulmonary disease, chronic hepatitis, and chronic renal insufficiency; (3) various serious diseases, such as liver failure, respiratory and circulatory failure, renal failure, severe acute pancreatitis; (4) endocrine and metabolic syndromes, such as thyroid dysfunction and diabetes mellitus; (5) autoimmune diseases presently taking glucocorticoids; (6) mental illness. Individual characteristics including sex, height, weight, age and diagnosis were acquired from the medical record system.

The retrospective study procedures were conducted in accordance with the Declaration of Helsinki and approved by the Ethics Committee of Beijing Youan Hospital, Beijing, China in February 2021 (LL-2021-018-K). Informed consent was waived because of the retrospective nature of the study and assurance of patient privacy.

### Assessment of skeletal muscle area

**FIGURE 1:**
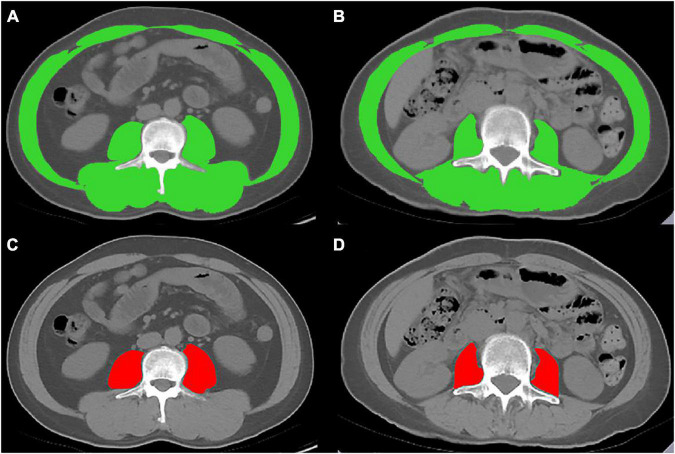
Cross-sectional computed tomographic images at the third lumbar vertebrae level. The skeletal muscle area (SMA) was measured by manual tracing in **(A)** male and **(B)** female. The psoas muscle area (PMA) was measured by manual tracing in **(C)** male and **(D)** female.

Abdominal non-contrast-enhanced CT scans were performed in a supine position using various multi-detector CT scanners (Supplementary Table 1). A transverse CT image at the level of the third lumbar vertebra (L3) was identified and exported from each scan by four abdominal radiologists (WS, CY, ZW, and LW, with more than 10 years of experience). And then measured by five experienced hepatologists (NG, YZ, and NLi from the Beijing Youan Hospital, Capital Medical University; QX from Nanyang First People’s Hospital; and WQ from the First Hospital of Lanzhou University, with more than 2 years of experience). Slice-O-Matic software (V5.0; Tomovision, Magog, Canada) was used to analyze the images using the –29 to 150 Hounsfield unit (HU; Figure 1) (11). The cross-sectional skeletal muscle area (including the psoas, erector spinae, quadratus lumborum, transversus abdominis, external and internal obliques, and rectus abdominis muscles) (SMA, cm^2^) and psoas muscle area (PMA, cm^2^) at the L3 level was calculated and then normalized for height squared (m^2^) to obtain the skeletal mass index (L3-SMI, m^2^/m^2^) and the psoas muscle mass index (L3-PMI, m^2^/m^2^).

All the images were reviewed by two observers to assess inter-observer reliability. To assess intra-observer reliability, images were measured by one observer (NG) twice.

### Statistical analysis

Statistical analyses were performed using SPSS 24.0 (IBM SPSS, Chicago, IL, USA). The continuous variables were shown as mean ± SD or median with an interquartile range. For categorical variables, data are presented as absolute or relative frequencies. Mann Whitney U-test and Kruskal Wallis test coupled with *post hoc* comparisons were used to testing the differences of continuous variables between groups. For the total population, the correlation between L3-PMI with age and L3-SMI were visualized with scatterplots and assessed using the Pearson correlation coefficient. Residual analysis was further validated for correlation between L3-PMI and L3-SMI. Low psoas muscle index was defined as the 5th percentile or two standard deviations below the mean value of the study group. Inter- and intra-observer agreement over CT scan readings was determined using the intraclass correlation coefficient (ICC). Statistical significance was defined as *p* < 0.05.

## Results

### Study population

This study included 1,787 healthy individuals (902 males, 885 females). The mean age of the study population was 45 ± 15 years old (range 20–88 years). The numbers of individuals per each age group were as follows: 354 (19.8%) for ages 20–29 years; 344 (19.3%) for ages 30–39 years; 363 (20.3%) for ages 40–49 years; 365 (20.4%) for ages 50–59 years; and 361 (20.2%) for ages over 60 years. The mean BMI of the study population was 23.52 ± 3.15 kg/m^2^. There was no significant difference in age between the male and female groups (44.9 vs 45.65 y, *p* = 0.297). Males showed significantly heavier weight, higher body mass index and skeletal muscle mass (*p* < 0.001). In addition, the mean L3-SMI and L3-PMI in males were significantly higher than that of females (*p* < 0.001). The baseline characteristics of the total population and sex-specific population were shown in Table 1. For CT scan readings, a high intra-observer (ICC = 1.000, *p* < 0.001) and inter-observer agreement (ICC = 0.996, *p* < 0.001) were observed.

**TABLE 1:** Characteristics of study population according to sex.

Variable	Total (*n* = 1,787)	Male (*n* = 902)	Female (*n* = 885)	*P*-value (Male vs. Female)
Age, y	45.2 ± 15.15	44.90 ± 15.20	45.65 ± 15.10	0.297
Height, m	1.68 ± 0.08	1.74 ± 0.06	1.62 ± 0.05	<0.001
Weight, kg	66.68 ± 11.18	71.99 ± 10.64	61.27 ± 8.91	<0.001
BMI, kg/m^2^	23.52 ± 3.15	23.79 ± 3.17	23.26 ± 3.12	<0.001
L3−SMA, cm^2^	125.61 ± 31.65	149.97 ± 23.99	100.78 ± 14.66	<0.001
L3−SMI, cm^2^/m^2^	43.99 ± 8.64	49.58 ± 7.45	38.29 ± 5.45	<0.001
L3−PMA, cm^2^	16.75 ± 6.19	20.96 ± 5.51	12.46 ± 3.15	<0.001
L3−PMI, cm^2^/m^2^	5.84 ± 1.86	6.93 ± 1.76	4.73 ± 1.17	<0.001

BMI, body mass index; L3-SMA, skeletal muscle area at the third lumbar vertebra; L3-SMI, skeletal muscle index at the third lumbar vertebra; L3-PMA, psoas muscle area at the third lumbar vertebra; L3-PMI, psoas muscle mass index at the third lumbar vertebra. Values are expressed as mean ± SD. Mann–Whitney U-test was used to compare the differences of continuous variables between sex groups.

### L3-PMI distributions are classified according to sex and age decades

**FIGURE 2:**
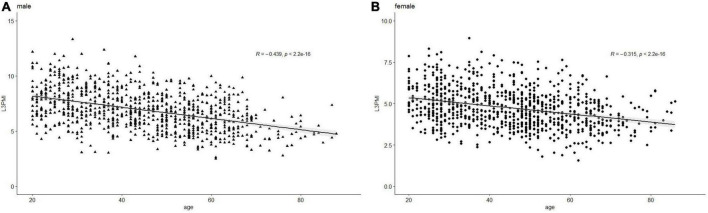
Scatter graph of the L3-PMI classified according to age decades in **(A)** male and **(B)** female. Data are analyzed using the Pearson correlation coefficient.

The characteristics of L3-PMI according to sex and age decades are shown in Table 2. The L3-PMI declined evidently as the age of the group increased, regardless of sex. Therefore, we analyzed L3-PMI distributions classified according to age decades. A significant negative relationship was observed between the L3-PMI and age decades in both males (*R* = –0.439, *p* < 0.001; Figure 2A) and females (*R* = –0.315, *p* < 0.001; Figure 2B).

**TABLE 2:** Characteristics of study population according to sex and age.

	Male					Female				
Age, y	20–29	30–39	40–49	50–59	≥60	20–29	30–39	40–49	50–59	≥60
Variable	(*n* = 185)	(*n* = 176)	(*n* = 181)	(*n* = 181)	(*n* = 179)	(*n* = 169)	(*n* = 168)	(*n* = 182)	(*n* = 184)	(*n* = 182)
Height, m	1.76 ± 0.05	1.75 ± 0.06	1.73 ± 0.06	1.74 ± 0.07	1.71 ± 0.06	1.63 ± 0.04	1.63 ± 0.05	1.62 ± 0.04	1.63 ± 0.06	1.60 ± 0.06
Weight, kg	70.70 ± 10.07	76.22 ± 11.88	72.99 ± 10.71	72.57 ± 9.56	67.56 ± 8.95	58.49 ± 8.16	60.90 ± 10.21	60.61 ± 6.95	63.92 ± 8.91	62.17 ± 9.22
BMI, kg/m^2^	22.80 ± 2.99	24.80 ± 3.63	24.32 ± 3.26	24.05 ± 2.68	22.99 ± 2.74	21.93 ± 3.02	22.87 ± 3.50	23.21 ± 2.44	23.99 ± 2.97	24.15 ± 3.10
L3−SMA, cm^2^	159.13 ± 22.15	158.83 ± 23.47	153.31 ± 22.90	147.35 ± 20.82	131.04 ± 18.75	102.57 ± 14.30	104.10 ± 15.71	102.92 ± 13.96	101.18 ± 13.94	93.53 ± 13.00
L3−SMI, cm^2^/m^2^	51.34 ± 7.00	51.73 ± 7.30	51.11 ± 7.16	49.05 ± 7.44	44.61 ± 5.92	38.47 ± 5.31	39.17 ± 5.78	39.42 ± 5.12	38.11 ± 5.72	36.35 ± 4.80
L3−PMA, cm^2^	24.77 ± 5.21	22.64 ± 5.55	21.04 ± 4.63	19.02 ± 4.27	17.27 ± 4.48	13.95 ± 3.30	13.34 ± 3.06	12.43 ± 2.99	11.91 ± 2.81	10.86 ± 2.66
L3−PMI, cm^2^/m^2^	8.01 ± 1.72	7.46 ± 1.65	7.01 ± 1.49	6.33 ± 1.48	5.89 ± 1.51	5.23 ± 1.22	5.02 ± 1.16	4.76 ± 1.13	4.48 ± 1.09	4.22 ± 0.98

BMI, body mass index; L3-SMA, skeletal muscle area at the third lumbar vertebra; L3-SMI, skeletal muscle index at the third lumbar vertebra; L3-PMA, psoas muscle area at the third lumbar vertebra; L3-PMI, psoas muscle mass index at the third lumbar vertebra. Values are expressed as mean ± SD.

### Relationship between L3-PMI and L3-SMI

**FIGURE 3:**
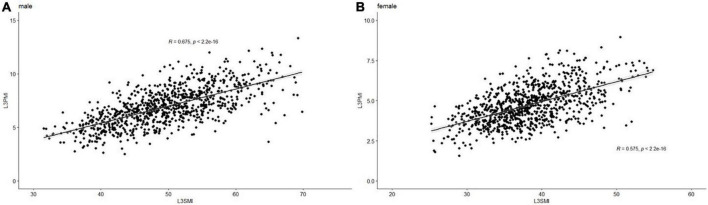
The relationship between L3-PMI and L3-SMI in **(A)** male and **(B)** female. Data are analyzed using the Pearson correlation coefficient.

**FIGURE 4:**
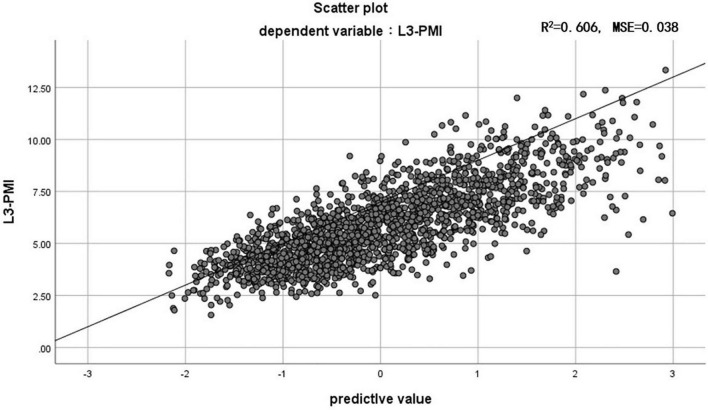
The relationship between L3-PMI and L3-SMI in the whole population. MSE, mean squared error. Data are analyzed using the residual analysis.

Previous research shows that the skeletal muscle index, taken at the third lumbar vertebra (L3-SMI, cm^2^/m^2^), has become widely recognized as an important indicator of evaluating total skeletal tissue (11). To determine whether the L3-PMI reflected the skeletal muscle mass, we calculated the L3-SMI in all subjects. The characteristics of L3-PMI and L3-SMI according to sex and age decades are also shown in Table 2. Results indicated that there was a strong correlation between L3-PMI and L3-SMI measured by CT, especially in males. As the scatter graph showed, there was a positive linear correlation between L3-PMI with L3-SMI. And the correlation was evident both in males (*R* = 0.675, *p* < 0.001; Figure 3A) and females (*R* = 0.575, *p* < 0.001; Figure 3B). According to Figure 4, 60.6% of the variation in L3-PMI is related to the variation in L3-SMI (*R*^2^ = 0.606), and the fit of L3-PMI with L3-SMI is better (MSE = 0.038). These results suggest that the L3-PMI can use as an alternative indicator of L3-SMI for the diagnosis of skeletal muscle mass loss in clinical practice.

### Reference values for L3-PMI

As previously described, the L3-PMI was affected by sex and age. We took these factors into consideration and calculated the sex-specific L3-PMI in each age group. The reference values of L3-PMI were defined as the 5th percentile or two standard deviations below the mean value of healthy individuals (Table 3). The reference values of L3-PMI in males were 5.41 cm^2^/m^2^ for 20–29 years, 4.71 cm^2^/m^2^ for 30–39 years, 4.65 cm^2^/m^2^ for 40–49 years, 4.10 cm^2^/m^2^ for 50–59 years and 3.68 cm^2^/m^2^ for over 60 years by using 5th percentile threshold. Similarly, the reference values of L3-PMI in females were 3.32 cm^2^/m^2^ for 20–29 years, 3.40 cm^2^/m^2^ for 30–39 years, 3.18 cm^2^/m^2^ for 40–49 years, 2.91 cm^2^/m^2^ for 50–59 years and 2.62 cm^2^/m^2^ for over 60 years. When using two standard deviations below the mean (mean-2SD) as the reference, the values for each age group were 4.57 cm^2^/m^2^, 4.16 cm^2^/m^2^, 4.03 cm^2^/m^2^, 3.37 cm^2^/m^2^, 2.87 cm^2^/m^2^ for males and 2.79 cm^2^/m^2^, 2.70 cm^2^/m^2^, 2.50 cm^2^/m^2^, 2.30 cm^2^/m^2^, 2.26 cm^2^/m^2^ for females, respectively (data not shown in Table 3).

**TABLE 3:** Reference values of L3-PMI (cm^2^/m^2^) according to sex and age decades by using mean-2SD and 5th percentile.

	Mean-2SD (cm^2^/m^2^)	5th percentile (cm^2^/m^2^)
Males (age, y)		
20–29	4.57	5.41
30–39	4.16	4.71
40–49	4.03	4.65
50–59	3.37	4.10
≥60	2.87	3.68
Females (age, y)		
20–29	2.79	3.32
30–39	2.70	3.40
40–49	2.50	3.18
50–59	2.30	2.91
≥60	2.26	2.62

L3-PMI, psoas muscle mass index at the third lumbar vertebra; SD, standard deviation.

## Discussion

Muscle mass and strength vary across a lifetime-generally increasing with the growth in youth, being maintained in midlife and then decreasing with aging (7). Loss of muscle mass and strength with aging is inevitable. While sarcopenia is mainly observed in older people, it can also develop in younger adults due to some chronic diseases, such as liver cirrhosis (12). Thus, sarcopenia can be classified as primary which is caused by aging itself, and secondary by some chronic diseases. In our study, 1,787 healthy adults were selected and divided into groups according to age. The sex and number of people in each group were similar.

So far, several methods can be used to assess skeletal muscle mass, such as DXA, CT, MRI and BIA (6). However, since BIA depends on stable hydration status, the accuracy is far from satisfactory (12). DXA and MRI are not routine clinical examination items, so it is difficult to carry out a retrospective study. CT was recommended as the gold standard for assessing muscle mass or quantity, because it can clearly separate the skeletal muscle from the body fat. Additionally, CT is commonly used to perform abdominal screenings in clinical diagnosis and physical examination. The skeletal muscle mass index taken at the third lumbar vertebrae (L3-SMI, cm^2^/m^2^) (the sum of the third cross-sectional areas of the right and left skeletal muscle/height squared) is one of the established methods for evaluating skeletal muscle mass and has been accepted to define sarcopenia in most countries, especially in western countries (13–15).

However, we find that L3-SMI has some disadvantages compared to L3-PMI. First, L3-SMI measures much more muscles than L3-PMI and includes some muscle groups with less regular shape, which leads to longer measurement time and potentially higher measurement errors. L3-PMI only needs to measure the psoas muscle (with regular shape and high recognition), which can significantly improve the measurement efficiency and accuracy no matter manual or software measurement. Secondly, L3-SMI has a weaker reflection on muscle mass in females than in males. A study showed reduced muscle density in female with cirrhosis compared to healthy individuals, but no significant difference in L3-SMI (14). Female muscle is infiltrated with higher amounts of fat at the early stages of sarcopenia (16), resulting in L3-SMI that does not reflect their muscle loss well. However, in our study, L3-PMI decreased significantly with age, regardless of sex, which is consistent with the physiological characteristics of skeletal muscle decreasing with age. In the previous study, we analyzed the relationship between L3-SMI and age (17). The L3-PMI in relation to age was stronger than that of L3-SMI. The L3-PMI varies significantly with age and has a strong sensitivity. Finally, L3-SMI was affected by body mass index (BMI). The Asian Working Group for Sarcopenia (AWGS) proposed to use BMI correction when using L3-SMI to diagnose sarcopenia in the 2019 Consensus Update on Sarcopenia Diagnosis and Treatment (18). However, we analyzed the correlation between L3-PMI and BMI in our study. The results show that the correlation is weak (Supplementary Figures 1, 2). When using L3-PMI to diagnose sarcopenia, BMI correction may not be required. The psoas muscle area might be a simple and practical measurement in diagnosing sarcopenia. Further study is needed to compare the ability of L3-PMI and L3-SMI by predicting adverse clinical outcomes.

Previous studies have proposed low L3-PMI reference values to define sarcopenia in the Asian population by CT from Japan, Korea, and Turkish. The AWGS and European Working Group on Sarcopenia in Older People (EWGSOP) have recommended mean minus two standard deviations or the 5th percentile as reference to define low skeletal muscle mass (2). The original studies defined L3-PMI reference values using one of the two methods above. Two studies reported PMI reference values calculated by mean minus two standard deviations. The first one took place in Japan in 2016 (6). Hamaguchi et al. defined donors aged between 20 and 49 as the reference group and calculated the L3-PMI reference values were 6.4 cm^2^/m^2^ for males and 3.9 cm^2^/m^2^ for females. Another one was in Korea in 2017 (19). Kim et al. defined age-specific reference values in healthy adults, which is similar to ours. One study reported L3-PMI reference values calculated by using the 5th percentile threshold in Turkish in 2019 (20). Based on the younger data (aged 20–40 years), they suggested that L3-PMI values less than 3.2 cm^2^/m^2^ for males and 2.87 cm^2^/m^2^ for females to define sarcopenia.

In recent studies, both the two methods above have been used to define the reference values. One is in Japan in 2020 (10). Ohara et al. analyzed the correlation between their two L3-PMI reference values and the Japan Society of Hepatology (JSH)-defined L3-SMI reference values by undertaking three evaluations of skeletal muscle mass simultaneously. As the L3-PMI reference values based on the lower 5th correlated better with the JSH L3-SMI reference values, they proposed reference values were 3.74 cm^2^/m^2^ for males and 2.29 cm^2^/m^2^ for females to define sarcopenia in adults under 50 years of age. Another one was in Turkish in 2021 (21). Bahat et al. defined the two sex-specific L3-PMI reference values, which were taken from 482 younger individuals aged between 18 and 40. But they did not specify which one to use. Comparison of studies investigating psoas muscle mass in different populations is shown in Table 4.

**TABLE 4:** Comparison of studies investigating psoas muscle mass in different population.

References	Country of study	Population	Individual numbers	Age range (years)	parameter	Cut-off methods	Cut-off value
Hamaguchi et al. (6)	Japan	Liver donors	541 (male: female = NA: NA)	20–49	L3-PMI	Mean-2SD	Men: 6.4 cm^2^/m^2^; Women: 3.9 cm^2^/m^2^
Kim et al. (19)	Korea	Adult patients who received abdominal CT in the health screening department	1,422 (male: female = 550:872)	20–89	L3-PMI	Mean-2SD	20–39 Men: 5.9 cm^2^/m^2^; Women: 4.0 cm^2^/m^2^ 40–49 Men: 4.7 cm^2^/m^2^; Women: 2.9 cm^2^/m^2^ 50–59 Men: 4.2 cm^2^/m^2^; Women: 2.4 cm^2^/m^2^ 60–69 Men: 3.7 cm^2^/m^2^; Women: 2.2 cm^2^/m^2^ 70–89 Men: 3.3 cm^2^/m^2^; Women: 1.5 cm^2^/m^2^
Furkan et al. (20)	Turkish	Kidney donors	270 (male: female = 134:136)	20–40	L3-PMI	5th Percentile	20–40 Men: 3.2 cm^2^/m^2^; Women: 2.9 cm^2^/m^2^
Ohara et al. (10)	Japan	Liver donors	293 (male: female = 163:130)	≤50	L3-PMI	5th Percentile	Men: 3.7 cm^2^/m^2^; Women: 2.3 cm^2^/m^2^
						Mean-2SD	Men: 3.3 cm^2^/m^2^; Women: 1.7 cm^2^/m^2^
Bahat et al. (21)	Turkish	Liver donors	482 (male: female = 268:214)	18–40	L3-PMI	5th Percentile	Men: 5.4 cm^2^/m^2^; Women: 3.6 cm^2^/m^2^
						Mean-2SD	Men: 4.6 cm^2^/m^2^; Women: 2.7 cm^2^/m^2^

L3-PMI, psoas muscle mass index at the third lumbar vertebra; CT, computed tomography; NA, not available; SD, standard deviation.

This is the first multicenter study to measure psoas muscle mass by CT at the L3 Level among healthy populations in northern China throughout a wide range of ages. The skeletal muscle mass taken at the third lumbar vertebrae provides an estimate of overall muscle mass and has been used in a number of studies to predict lean muscle mass (13). In our study, correlation with L3-SMI data gives reliability that the simple measurement of L3-PMI may represent the whole-body skeletal muscle mass.

In our study, age-specific reference values of L3-PMI used to define low muscle mass was established by two widely used methods, the 5th percentile and 2 standard deviations below the mean muscle mass of the study group, for comparison with the population in other studies. We found the two reference values were different evidently, and the reference values are lower by using mean-2SD than that using lower 5th. We are inclined to use the lower 5th to define sarcopenia, because it can improve the diagnosis sensitivity. However, we need more studies that specifically focus on the association of sarcopenia defined comparatively by the two methods above with adverse outcomes, to decide which reference values are better.

The main limitation of the present study is the retrospective study with insufficient information on other factors affecting skeletal muscle mass, such as exercise and eating habits. And the population of this study may not fully represent the healthy population due to sampling methods of only hospital patients, some of whom which may not be in great health despite not having serious chronic diseases. However, there is no doubt that they are relatively healthy. In the next step, more prospective studies should be conducted to explore the relationship between these reference values and certain clinical outcomes or diseases, such as liver diseases. Further research is needed to test the prognostic role of the reference values.

In conclusion, we defined the reference values of age-specific low skeletal muscle mass when simply evaluated by L3-PMI using CT images, which were taken from 1,787 healthy individuals aged between 20 and 88. The 5th percentile and mean-2SD were used to define low muscle mass. Further studies about the association of sarcopenia using these reference values with certain clinical outcomes or diseases are needed.

## Data availability statement

The original contributions presented in this study are included in the article/Supplementary material, further inquiries can be directed to the corresponding authors.

## Ethics statement

The retrospective study was approved by the Ethics Committee of Beijing Youan Hospital (LL-2021-018-K). We only obtained body composition data and relevant clinical information, and not involved the privacy of participants. The Ethics Committee did not require participants to sign informed consent.

## Author contributions

ZD, YC, LM, and MK conceived and designed the project. YC and ZD obtained funding. NG, SL, YZ, NLi, YP, ZH, RG, NLu, QX, and WQ contributed to data collection. WS, CY, ZW, and LW operated software. MX analyzed and interpreted data. MK, NLi, and LW drafted the manuscript. YC, ZD, DS, JL, and EC revised the manuscript. All authors approved the final version of the manuscript.
