# Corrigendum: Age-specific reference values for low psoas muscle index at the L3 vertebra level in healthy populations: A multicenter study

**DOI:** 10.3389/fnut.2023.1138289

**Published:** 2023-01-24

**Authors:** Ming Kong, Ning Lin, Lili Wang, Nan Geng, Manman Xu, Shanshan Li, Wenyan Song, Ying Zhou, Yuetong Piao, Zuoqing Han, Rong Guo, Chao Yang, Nan Luo, Zhong Wang, Quanxiao Xu, Daimeng Shi, Wanchun Qiu, Junfeng Li, Eddie C. Cheung, Lei Ma, Yu Chen, Zhongping Duan

**Affiliations:** ^1^Beijing Municipal Key Laboratory of Liver Failure and Artificial Liver Treatment Research, Fourth Department of Liver Disease, Beijing Youan Hospital, Capital Medical University, Beijing, China; ^2^Department of Radiology, The First Hospital of Lanzhou University, Lanzhou, China; ^3^Department of Radiology, Beijing Youan Hospital, Capital Medical University, Beijing, China; ^4^Department of Gastroenterology and Hepatology, The Chinese People's Liberation Army Rocket Force Characteristic Medical Center, Postgraduate Training Base of Jinzhou Medical University, Beijing, China; ^5^Department of Infection, Dalian Medical University, Dalian, China; ^6^Department of Radiology, The Second Hospital of Dalian Medical University, Dalian, China; ^7^Department of Infection, The Second Hospital of Dalian Medical University, Dalian, China; ^8^Diagnosis and Treatment Center of Hepatobiliary Diseases, Nanyang First People's Hospital, Nanyang, China; ^9^Department of Infection, The First Clinical Medical School of Lanzhou University, Lanzhou, China; ^10^Department of Infectious Diseases, Institute of Infectious Diseases, The First Hospital of Lanzhou University, Lanzhou, China; ^11^Division of Gastroenterology, School of Medicine, University of California, Davis, Davis, CA, United States; ^12^Center for Digestive Disease, The Seventh Affiliated Hospital, Sun Yat-sen University, Shenzhen, China

**Keywords:** skeletal muscle mass, sarcopenia, computed tomography, lumbar 3 vertebra, reference values, healthy adults

In the published article, there was an error in affiliation 12. Instead of “Center for Digestive Disease, The Seventh Affiliated Hospital, Sun Yat-sen University, Guangzhou, China,” it should read “Center for Digestive Disease, The Seventh Affiliated Hospital, Sun Yat-sen University, Shenzhen, China.”

In the published article, there was an error regarding the affiliation for Lili Wang. Instead of “Diagnosis and Treatment Center of Hepatobiliary Diseases, Nanyang First People's Hospital, Nanyang, China,” it should read “Department of Radiology, The First Hospital of Lanzhou University, Lanzhou, China.”

There was an error regarding the affiliation for Lei Ma. Instead of “Department of Infection, The Second Hospital of Dalian Medical University, Dalian, China,” it should read “Diagnosis and Treatment Center of Hepatobiliary Diseases, Nanyang First People's Hospital, Nanyang, China.”

The authors apologize for this error and state that this does not change the scientific conclusions of the article in any way. The original article has been updated.

