# Supplementary material for: Age-specific reference values for low psoas muscle index at the L3 vertebra level in healthy populations: A multicenter study
Source: Front Nutr. 2022 Dec 16;9:1033831. doi: 10.3389/fnut.2022.1033831 (PMC9800856; doi:10.3389/fnut.2022.1033831)
Supplement: Supplementary file 1 [file Data_Sheet_1.docx]

**Age-Specific Reference Values for Low Psoas Muscle Index at The L3 Vertebra Level in Healthy Populations: A Multicenter Study.**

Ming Kong, Ning Lin, Lili Wang

Supplementary Table 1. CT units used and detailed technical parameters

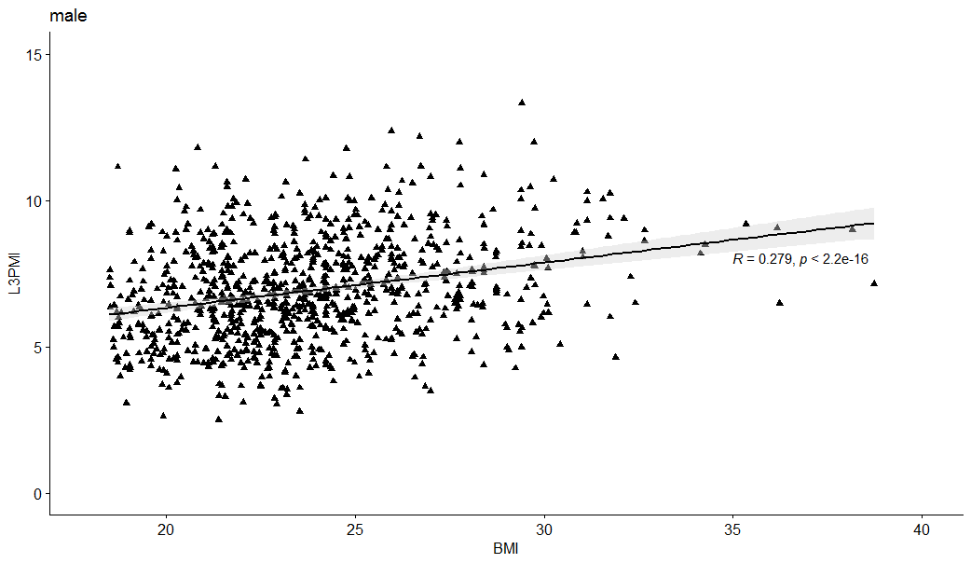

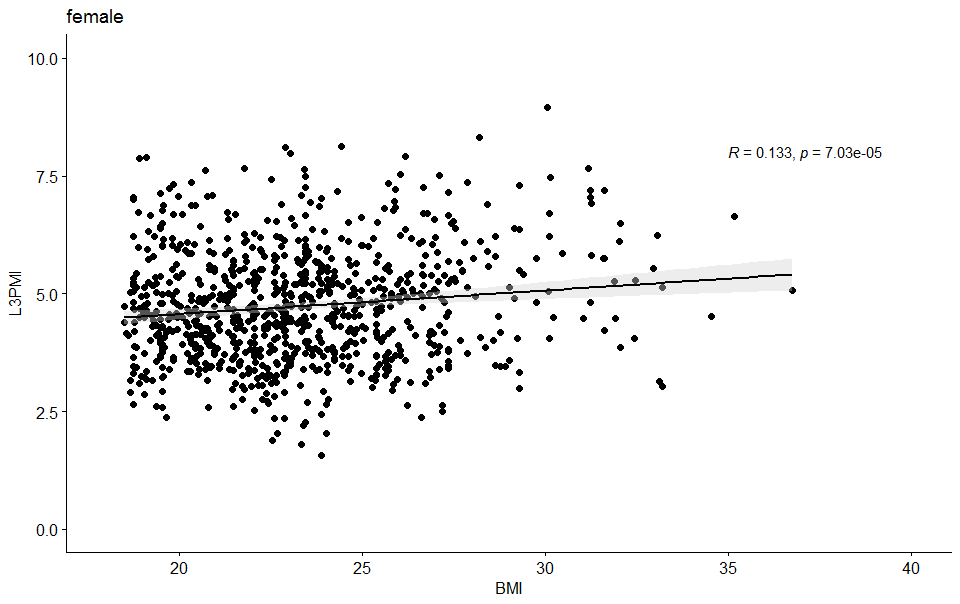


**B**

**A**

Supplementary Figure 1. The relationship between L3-PMI and BMI in (A) male and (B) female. Data are analyzed

using the Pearson correlation coefficient.


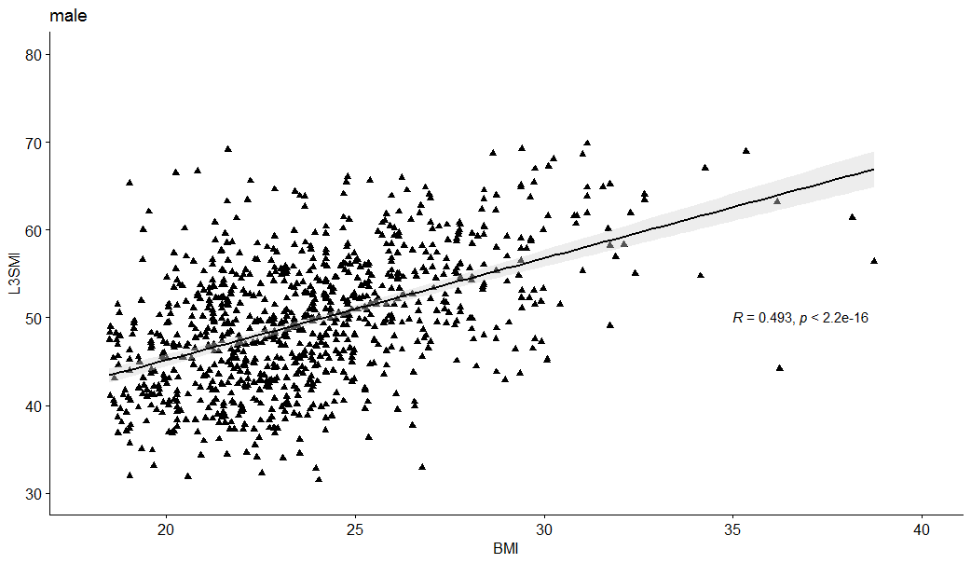

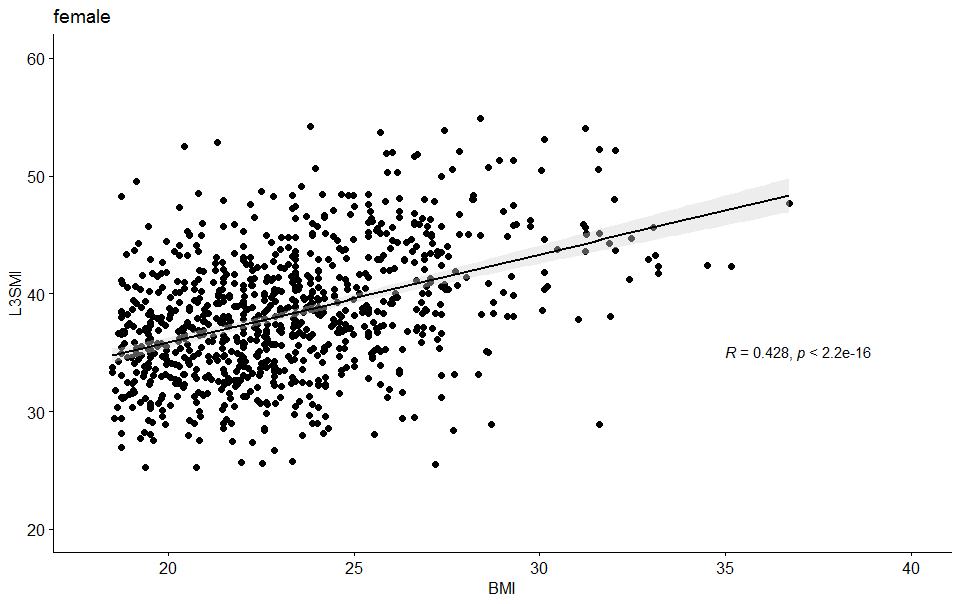


**B**

**A**

Supplementary Figure 2. The relationship between L3-SMI and BMI in (A) male and (B) female. Data are analyzed

using the Pearson correlation coefficient.
